# Intra- and peritumoral MRI radiomics assisted in predicting radiochemotherapy response in metastatic cervical lymph nodes of nasopharyngeal cancer

**DOI:** 10.1186/s12880-023-01026-1

**Published:** 2023-05-30

**Authors:** Hao Xu, Ai Wang, Chi Zhang, Jing Ren, Peng Zhou, Jieke Liu

**Affiliations:** Department of Radiology, Sichuan Clinical Research Center for Cancer, Sichuan Cancer Hospital & Institute, Sichuan Cancer Center, Affiliated Cancer Hospital of University of Electronic Science and Technology of China, Chengdu, China

**Keywords:** Nasopharyngeal cancer, Magnetic resonance imaging, Radiomics

## Abstract

**Background:**

To establish and validate radiomic models combining intratumoral (Intra) and peritumoral (Peri) features obtained from pretreatment MRI for the prediction of treatment response of lymph node metastasis from nasopharyngeal cancer (NPC).

**Methods:**

One hundred forty-five NPC patients (102 in the training and 43 in the validation set) were retrospectively enrolled. Radiomic features were extracted from Intra and Peri regions on the metastatic cervical lymph node, and selected with the least absolute shrinkage and selection operator (LASSO). Multivariate logistic regression analysis was applied to build radiomic models. Sensitivity, specificity, accuracy, and the area under the curve (AUC) of receiver operating characteristics were employed to evaluate the predictive power of each model.

**Results:**

The AUCs of the radiomic model of Intra, Peri, Intra + Peri, and Clinical-radiomic were 0.910, 0.887, 0.934, and 0.941, respectively, in the training set and 0.737, 0.794, 0.774, and 0.783, respectively, in the validation set. There were no significant differences in prediction performance among the radiomic models in the training and validation sets (all *P* > 0.05). The calibration curve of the radiomic model of Peri demonstrated good agreement between prediction and observation in the training and validation sets.

**Conclusions:**

The pretreatment MRI-based radiomics model may be useful in predicting the treatment response of metastatic lymph nodes of NPC. Besides, the generalization ability of the radiomic model of Peri was better than that of Intra and Intra + Peri.

**Supplementary Information:**

The online version contains supplementary material available at 10.1186/s12880-023-01026-1.

## Background

Nasopharyngeal cancer (NPC) is the most frequent type of head and neck cancer in Southeast Asia and South China [1–3]. NPC has a higher incidence (86.4%) of cervical lymph node metastasis [4]. Intensity-modulated radiotherapy (IMRT) is the primary treatment regimen for NPC. Additional chemotherapy is often administered in the treatment of patients with advanced stages because it has been shown to increase the overall survival rate [5–7]. Therefore, concurrent chemoradiotherapy (CCRT) has become the standard treatment for stage II-IVA NPC by the guidelines of the National Comprehensive Cancer Network (NCCN) [8]. Tumor response to radiochemotherapy is an independent prognostic factor for survival in NPC [9]. However, not all patients respond well to radiochemotherapy. Predicting the response to radiochemotherapy may result in more targeted and personalized treatment for NPC patients, avoiding unnecessary side effects and costs. However, there are no established biomarkers, and some have been proposed as research tools. Thus, there is an urgent need to identify an effective predictor for predicting treatment response in patients with NPC.

The ability of multimodality imaging biomarkers generated from computed tomography (CT), ^18^ F-fluorodeoxyglucose positron emission tomography, or MRI-DWI for the prediction of response to radiochemotherapy in NPC has been demonstrated [10–12]. MRI plays a vital role in NPC diagnosis and treatment management. MRI images, with a high soft tissue resolution, not only contain anatomical information about the primary NPC lesion and its adjacent constructions but also reflect the intra-tumor characteristics [13]. Radiomics is a rapidly emerging field that refers to the mining of quantitative features from a large number of medical images. It is widely used in disease identification, differential diagnosis, prognosis prediction, and treatment response evaluation [13–17]. According to recent studies, pretreatment MRI-based radiomics could predict progression-free survival in NPC [18, 19]. While lacking independent validation, the pretreatment MRI radiomic signature may predict early treatment response to initiation chemotherapy in NPC [20]. Peng et al. [9] discovered that tumor response to radiochemotherapy was recognized as an independent prognostic factor of 4-year disease-free, overall, and locoregional relapse-free survival. In another study, Liu et al. [21] revealed that an unsatisfactory tumor response (stable disease or disease progression) after neoadjuvant chemotherapy serves as a predictor of poor prognosis for advanced-stage NPC patients. However, these radiomics studies focused on the primary tumor and the intratumoral (Intra) region alone, without considering the utility of the peritumoral (Peri) microenvironment. Few studies have demonstrated the feasibility of combining the Intra and Peri radiomic features in the evaluation of the treatment response of metastatic lymph nodes in NPC.

Thus, our study aimed to evaluate the ability of pretreatment MRI-based radiomic models combining the Intra and Peri features for the prediction of treatment response of lymph node metastasis from NPC. We present the following article in accordance with the Multivariable Prediction Model for Individual Prognosis or Diagnosis (TRIPOD) reporting checklist [22].

## Materials and methods

### Patients

**Fig. 1:**
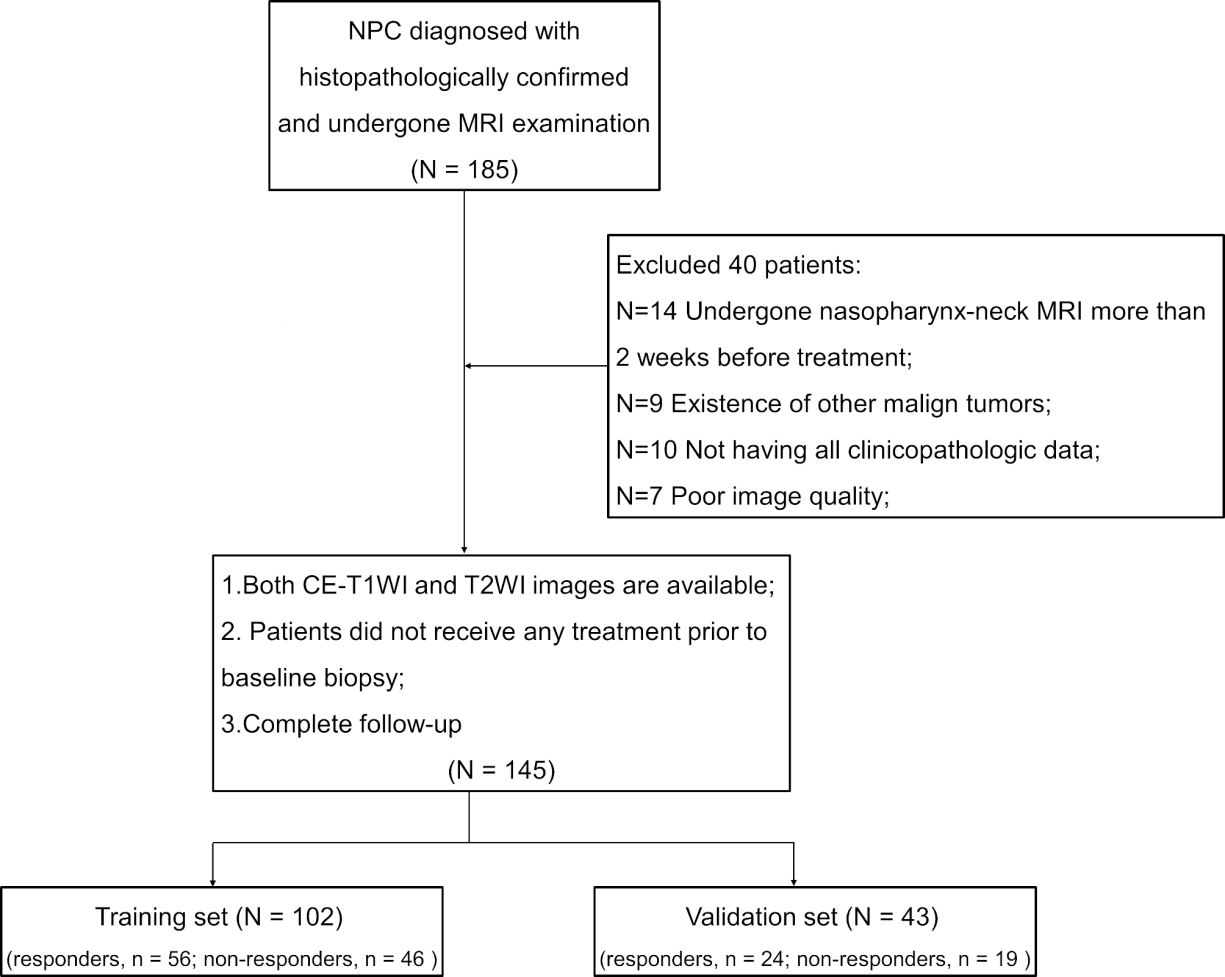
Flowchart shows patient selection. NPC, nasopharyngeal cancer; CE-T1WI, contrast-enhanced T1-weighted imaging; T2WI, T2-weighted imaging

The retrospective study received approval from our ethics committee and institutional review board, and informed consent was not required. Between October 2016 and November 2019, we enrolled 185 patients with NPC who received induction chemotherapy (IC) and CCRT. All patients received an individualized treatment based on NCCN guidelines [23]. The details of treatment regimens are shown in Additional file 1 (Additional file 1: S1). The inclusion criteria were as follows: (1) Primary tumor confirmed by biopsy as NPC; (2) available pretreatment T2-weighted imaging (T2WI) and contrast-enhanced T1-weighted imaging (CE-T1WI) images after biopsy; (3) available post-treatment T2WI and CE-T1WI images for predicting treatment response; (4) no treatment before baseline biopsy; and (5) available clinical variables such as age, sex, T-stage, N-stage, clinical stage, lymph node involvement, and lymph node gross tumor volume (GTV-ln). The exclusion criteria were as follows: (1) the period between baseline MRI and initial treatment was further than 2 weeks (n = 14); (2) the existence of other malign tumors (n = 9); (3) missing clinicopathological information (n = 10); (4) poor image quality (n = 7). Finally, a total of 145 consecutive patients (mean age 47.2 ± 12.6, range 13–77, male = 117, female = 28, stage II = 2, stage III = 59, stage IV = 84) were enrolled and allocated to the training set (102 patients, October 2016 to December 2018) and validation set (43 patients, December 2018 to November 2019). [22] (Fig. 1). The time all patients who underwent MRI examination after the completion of treatment was 2–14 weeks. Patients were staged based on the 8th version American Joint Committee on Cancer (AJCC) Tumor-Node-Metastasis (TNM) staging system [24]. Demographic information and stages were extracted from the Hospital Information System of our institution.

### MR images acquisition protocol

All patients underwent nasopharyngeal and cervical region MRI examination using a 1.5-T MR scanner (Avanto, Siemens, Germany) or a 3.0-T MR imaging scanner (Skyra, Siemens, Germany) with head-neck combined coils. Overall, 94/145 (64.8%) patients underwent imaging in the 1.5-T MRI scanner, and 51/145 (35.2%) underwent imaging in the 3.0-T scanner. To keep away from magnetic exposure and motion artifacts, patients were instructed to eliminate all metal-containing items and lie supinely in the scanner before scanning. Before the MRI examination, all patients will be required to wear earplugs and headphones to decrease noise. The DICOM format images of axial fat-suppressed CE-T1WI (contrast agent = Gd-DTPA, Magnevist, Schering, Berlin, Germany; dose = 0.1 mmol/kg body weight) and T2WI for each case were retrieved from the picture archiving and communication system (Carestream, Ontario, Canada). The details of MRI scanning parameters are shown in Additional file 1 (Additional file 1: S2). MRI images were reconstructed using the inverse fourier transform with the linear filling algorithm.

### Evaluation of lymph nodes

Multiple radiologic criteria were used to determine whether metastatic cervical lymph nodes were involved [25–28]. These included: (1) regions of central necrosis/cystic necrosis (T2WI with a focal high signal intensity or CE-T1WI with low signal intensity/ with or without an adjacent border of enhancement), (2) extracapsular distribution in any size lymph node, including ambiguous nodal boundaries, irregular nodal capsular improvement, and infiltration into neighboring muscle or fat, (3) the shortest diameter of the cervical or medial retropharyngeal lymph node is 10 mm; the lateral retropharyngeal lymph node is 5 mm.

In each patient, only the largest metastatic lymph node within the scanning range is considered. The largest lymph nodes were then assessed on T2WI for maximum axial diameter and minimum axial diameter long axis. The minimum axial diameter matched the node’s largest diameter in the axial plane perpendicular to its maximum axial diameter. The Response Evaluation Criteria in Solid Tumors 1.1 (RECIST) was then used to assess the largest lymph node’s response after treatment [29]. The responders were defined as a reduction of at least 30% in the largest lymph node’s maximum axial diameter. In contrast, non-responders had inadequate shrinking to qualify for the responders.

### Preprocessing of images

All pretreatment MRI images were transmitted to the Artificial Intelligence Kit software (A.K., version 3.2.0, GE Healthcare, China) for preprocessing. Firstly, all raw images were reconstructed using the trilinear interpolation algorithm to a final voxel size of 1 mm ×1 mm × 1 mm. The center was employed to align the interpolation grid’s location, and its measurements were rounded to the closest integer. Secondly, the Gaussian filter and bias-field correction were applied after the reconstruction. Lastly, to eliminate the influence of the different ranges of gray values, image was normalized using z-score normalization.

### Image segmentation

**Fig. 2:**
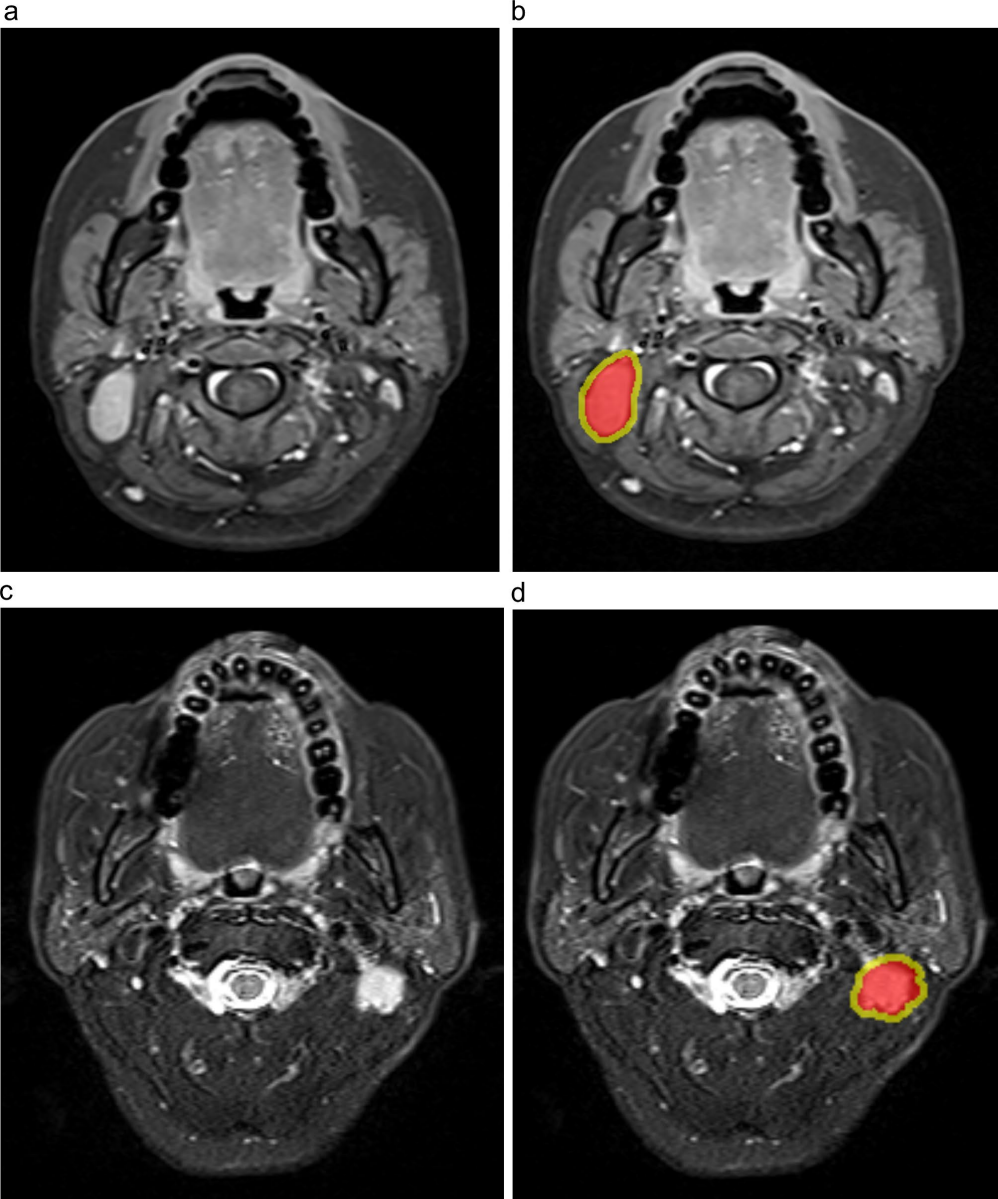
Representative slice of the MRI images and the corresponding Intra and Peri regions of interest (ROIs). The Intra ROI (red regions) drawn by a junior radiologist with the Peri ROI (yellow regions) generated by equidistant 3-dimensional dilation of the Intra regions with 2 mm. (**a, b**) Axial pretreatment CE-T1WI images. (**c, d**) Axial pretreatment T2WI images

Three-dimensional manual segmentation of the largest lymph nodes and region of interest (ROIs) were delineated by two radiologists with 7 and 20 years of clinical diagnosing experience using ITK-SNAP software (version 3.8.0, http://www.itksnap.org) to generate Intra ROIs. Both radiologists were blinded to the clinical information. The ROIs based on the lymph nodes were drawn to cover the whole tumor on each consecutive slice of the T2WI and CE-T1WI images separately, necrosis and extranodal extension regions were avoided when delineated ROIs. A distance of 2 mm from the tumor boundary was defined as the Peri region. The Peri ROI was then obtained by subtracting the Intra ROI from the dilated ROI. Figure 2 showed a typical MRI image and its associated Intra and Peri ROIs. The reproducibility of radiomic features was evaluated using the intraclass correlation coefficient (ICC). The senior radiologist (PZ) re-delineated 30 lymph nodes that were randomly selected from the T2WI and CE-T1WI images, respectively. Feature with an ICC > 0.75 was regarded as having good reproducibility and remained.

### Feature extraction and selection

Radiomic features from the Intra and Peri ROIs were extracted using an open-source Python software (PyRadiomics, version 3.0, https://pyradiomics.readthedocs.io) [30] that followed the image biomarker standardisation initiative (IBSI) standard [31]. These features belonged to three categories: shape, firstorder, and texture features. Image intensity was discretized using a fixed bin width of 25. Detailed descriptions of these features are provided in Table S1 and Table S2 in the Additional file 1. Radiomic features were standardized using z-score normalization. Five procedures were performed to select radiomic features. We used the Mann-Whitney U test to make the initial selection from the training set. For the remaining significant features, the P-value threshold was set at 0.05. Then, to eliminate redundant features, Spearman correlation assessment and maximum relevance-minimum redundancy (mRMR) were performed successively. Features with Spearman correlation coefficient values greater than 0.9 were eliminated. 15 features with high relevance and low redundancy were retained after mRMR. Finally, the least absolute shrinkage and selection operator (LASSO) algorithm and multivariate logistic regression using Akaike information criterion as the stopping rule were applied to select the most predictive features [32, 33].

### Radiomic model building

Radiomic models (Intra, Peri, and Intra + Peri) were built using the radiomics score (Rad-score). The Rad-score was computed utilizing a linear combination of the chosen features weighted by their corresponding coefficients. To examine possible multicollinearity between the features included in the radiomic model, Spearman’s correlations were employed.

### Clinical-radiomic model construction

Univariate logistic regression analysis was utilized to recognize independent risk factors for differentiating responders from non-responders between radiomic models and clinical variables. Subsequently, a clinical-radiomic model combining the radiomic model and significant independent risk factors was built in the training set, using the multivariable logistic regression analysis.

### Model validation

The predictive performance of the radiomic model and clinical-radiomic model were assessed in the training set and then validated in the validation set using the area under the receiver operating characteristic curve (ROC-AUC). The threshold was established utilizing the maximum Youden index (sensitivity + specificity − 1) on the training set and this same threshold was used for the validation set. We also calculated the accuracy, sensitivity, and specificity. The 95% confidence intervals (CIs) were calculated using the exact binomial method. Calibration curves were plotted to evaluate the calibration performance of radiomic models. The Hosmer-Lemeshow (H-L) test was used to assess the goodness-of-fit of radiomic models [34].

### Statistical analysis

SPSS (version 26.0, available at https://www.ibm.com), MedCalc (version 18.2.1, available at https://www.medcalc.org), and R software (version 4.0.0, http://www.r-project.org) were utilized for all statistical analyses. The Shapiro-Wilk test was used to examine if quantitative data had a normal distribution. The clinical variables of the responders and non-responders were compared using an independent samples t-test, the Mann-Whitney U test, Fisher’s exact analysis, or the chi-squared (χ2) test, as applicable. The DeLong test was used to compare the AUCs among the radiomic models [35]. *P* < 0.05 was considered statistically significant (two-tailed).

## Results

### Baseline characteristics of the patient

The clinical characteristics and sociodemographics of 145 patients are listed in Table 1. The numbers of responders and non-responders in the training and validation sets were 56 and 46, and 24 and 19, respectively. Among the involved population, the numbers of metastatic retropharyngeal lymph nodes and cervical lymph nodes in the training and validation sets were 24 and 78, and 6 and 37, respectively. There were no significant differences in age, sex, T- stage, N-stage, clinical stage, and lymph node involvement between responders and non-responders in the training and validation sets (all *P* > 0.05). There was a significant difference in the GTV-ln between responders and non-responders in the two sets (*P* < 0.001 and = 0.047).

**Table 1:** Clinical characteristics of patients in the training and validation sets

Characteristics	Training set (n = 102)			Validation set (n = 43)		
	Responders (n = 56)	Non-responders (n = 46)	*P*	Responders (n = 24)	Non-responders (n = 19)	*P*
Age (mean ± SD)	45.4 ± 12.6	48.0 ± 13.4	0.310	47.5 ± 11.1	50.0 ± 12.4	0.492
Sex, n (%)			0.825			0.708
Female	10 (17.9)	9 (19.6)		6 (25.0)	3 (15.8)	
Male	46 (82.1)	37 (80.4)		18 (75.0)	16 (84.2)	
T-stage, n (%)			0.559			1.000
T1	6 (10.7)	2 (4.3)		1 (4.2)	0 (0.0)	
T2	16 (28.6)	16 (34.8)		5 (20.8)	4 (21.1)	
T3	19 (33.9)	13 (28.3)		13 (54.2)	11 (57.9)	
T4	15 (26.8)	15 (32.6)		5 (20.8)	4 (21.0)	
N-stage, n (%)			0.207			1.000
N1	4 (7.1)	0 (0.0)		1 (4.2)	1 (5.3)	
N2	30 (53.6)	28 (60.9)		13 (54.1)	11 (57.9)	
N3	22 (39.3)	18 (39.1)		10 (41.7)	7 (36.8)	
Clinical stage, n (%)			0.600			0.606
II	2 (3.6)	0 (0.0)		0 (0.0)	0 (0.0)	
III	20 (35.7)	16 (34.8)		12 (50.0)	11 (57.9)	
IV	34 (60.7)	30 (65.2)		12 (50.0)	8 (42.1)	
LN involvement, n (%)			0.136			0.757
Retropharyngeal LN	10 (17.9)	14 (30.4)		3 (12.5)	3 (15.8)	
Cervical LN	46 (82.1)	32 (69.6)		21 (87.5)	16 (84.2)	
GTV-ln (Gy), median (IQR)	52.80 (46.20–60.00)	45.60 (36.07–52.35)	< 0.001*	51.50 (42.07–54.22)	41.80 (33.00–52.30)	0.047*

LN lymph node, GTV-ln Lymph node gross tumor volume, IQR interquartile range. * indicates statistical significant difference

### Feature selection

5408 radiomic features were extracted (2704 in the Intra region and 2704 in the Peri region). After calculating the ICCs, the numbers of reproducible features (ICCs > 0.75) from the CE-T1WI and T2WI images in the Intra region and Peri region were 940 and 1076, and 971 and 975, respectively. The detailed feature selection process and LASSO results are shown in Additional file 1 (Additional file 1: Fig. S1 and Fig. S2). Finally, we selected 4, 4, and 5 features for Intra, Peri, and Intra + Peri radiomic models construction (Additional file 1: Table S3 and Table S4). In Additional file 1 (Additional file 1: Table S5), we presented the mean and standard deviation of the features.

### Radiomic model development

The radiomic model of Intra was constructed using the following formula: Rad-score = -0.8765–0.8685 × CE-T1WI_firstorder_wavelet.HHH.Skewness + 1.2215 × CE-T1WI_GLDM_wavelet.HHL.LDLGLE + 2.8194 × T2WI_GLCM_LoG.sigma.1.5.mm.3D.ClusterShade – 2.1866 × T2WI_GLCM_wavelet.LLH.MaximumProbability.

The radiomic model of Peri was constructed using the following formula: Rad-score = -0.0330–1.3038 × CE-T1WI_firstorder_LoG.sigma.0.5.mm.3D.Minimum + 1.8829 × CE-T1WI_GLRLM_wavelet.HHH.SRHGLE + 1.1131 × T2WI_GLDM_wavelet.LHL.SDHGLE – 1.3508 × T2WI_GLCM_wavelet.LLH.ClusterTendency.

The radiomic model of Intra + Peri was constructed using the following formula: Rad-score = -0.0335–1.0301 × Intra_CE-T1WI_firstorder_LoG.sigma.0.5.mm.3D.90Percentile – 3.0175 × Intra_T2WI_GLCM_wavelet.LLH.MaximumProbability – 1.8770 × Peri_CE-T1WI_firstorder_LoG.sigma.0.5.mm.3D.Minimum + 1.3759 × Peri_CE-T1WI_GLRLM_wavelet.HHH. SRHGLE + 1.4714 × Peri_T2WI_GLRLM_wavelet.HLL.LRLGLE.

The distribution of the Rad-score of responders and non-responders in the training and validation sets is shown in Additional file 1 (Additional file 1: Fig. S3). Additional file 1 (Additional file 1: Table S6) provided detailed spearman’s correlation values between the selected features in the radiomic models.

### Clinical-radiomic model construction

**Fig. 3:**
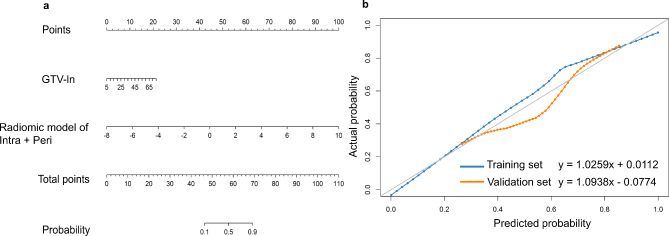
(**a**) The Clinical-radiomic model was developed by integrating the radiomic model of Intra + Peri and GTV-ln in the training set. The different values of each variable corresponds to a point at the top of the graph, while the sum of points of all variables corresponds to a total point. Drawing a line from the total points to the bottom line is the probability of responders. (**b**) Calibration curves of the Clinical-radiomic model for predicting treatment response in the training and in the validation sets, respectively. Intra, intratumoral; Peri, peritumoral; GTV-ln, Lymph node gross tumor volume

Univariate logistic regression analysis found that GTV-ln was associated with responders (OR = 1.055, *P* = 0.003). Further multivariate logistic regression analysis found that only GTV-ln in the Intra + Peri dataset was associated with responders (*P* = 0.036) (Table 2). Therefore, the Clinical-radiomic model was developed by integrating the Intra + Peri_Rad-score and GTV-ln (Fig. 3a). The clinical-radiomic model was constructed using the following formula: Clinical-radiomic model = -2.8790 + 0.0590 × GTV-ln + 1.0770 × Intra + Peri_Rad-score.

**Table 2:** Univariate and multivariate logistic regression analyses for predictive factors of responders in the training set

Variables		Odds ratio	95% CI	*P*
Univariate logistic analysis				
GTV-ln		1.055	1.018–1.093	0.003
Intra_Rad-score		2.718	1.857–3.980	< 0.001
Peri_Rad-score		2.718	1.837–4.022	< 0.001
Intra + Peri_Rad-score		2.718	1.835–4.026	< 0.001
Multivariate logistic analysis				
Intra clinical-radiomic	GTV-ln	1.041	0.992–1.091	0.102
	Rad-score	2.577	1.784–3.723	< 0.001
Peri clinical-radiomic	GTV-ln	1.039	0.994–1.086	0.087
	Rad-score	2.638	1.770–3.932	< 0.001
Intra + Peri clinical-radiomic	GTV-ln	1.061	1.004–1.121	0.036
	Rad-score	2.935	1.867–4.613	< 0.001

GTV-ln Lymph node gross tumor volume, Intra intratumoral, Peri peritumoral

### Performance and validation of predicting model

**Fig. 4:**
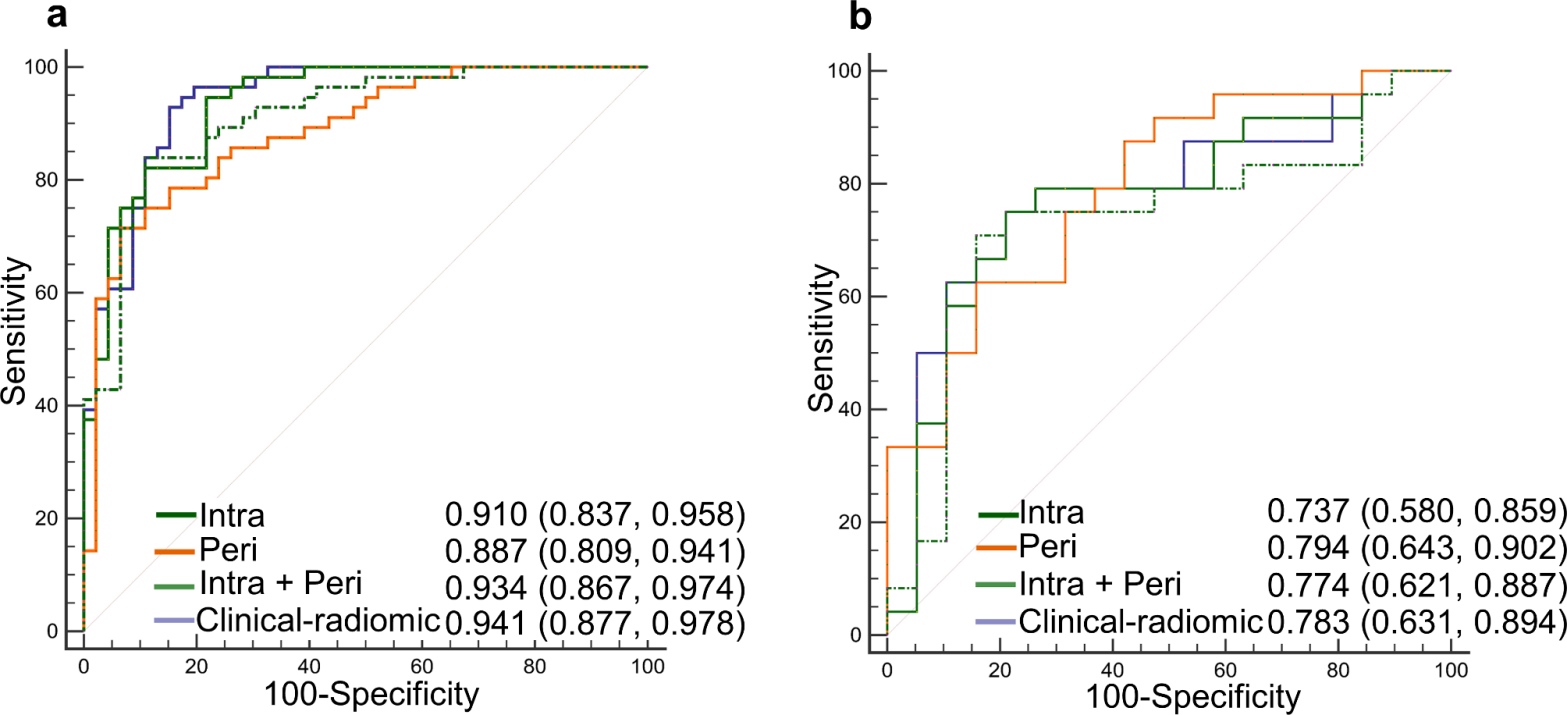
Receiver operating characteristic curves of radiomic models for predicting treatment response in the training set (**a**) and validation set (**b**), respectively. The numbers in parentheses are the 95% confidence interval. Intra, intratumoral; Peri, peritumoral

**Fig. 5:**
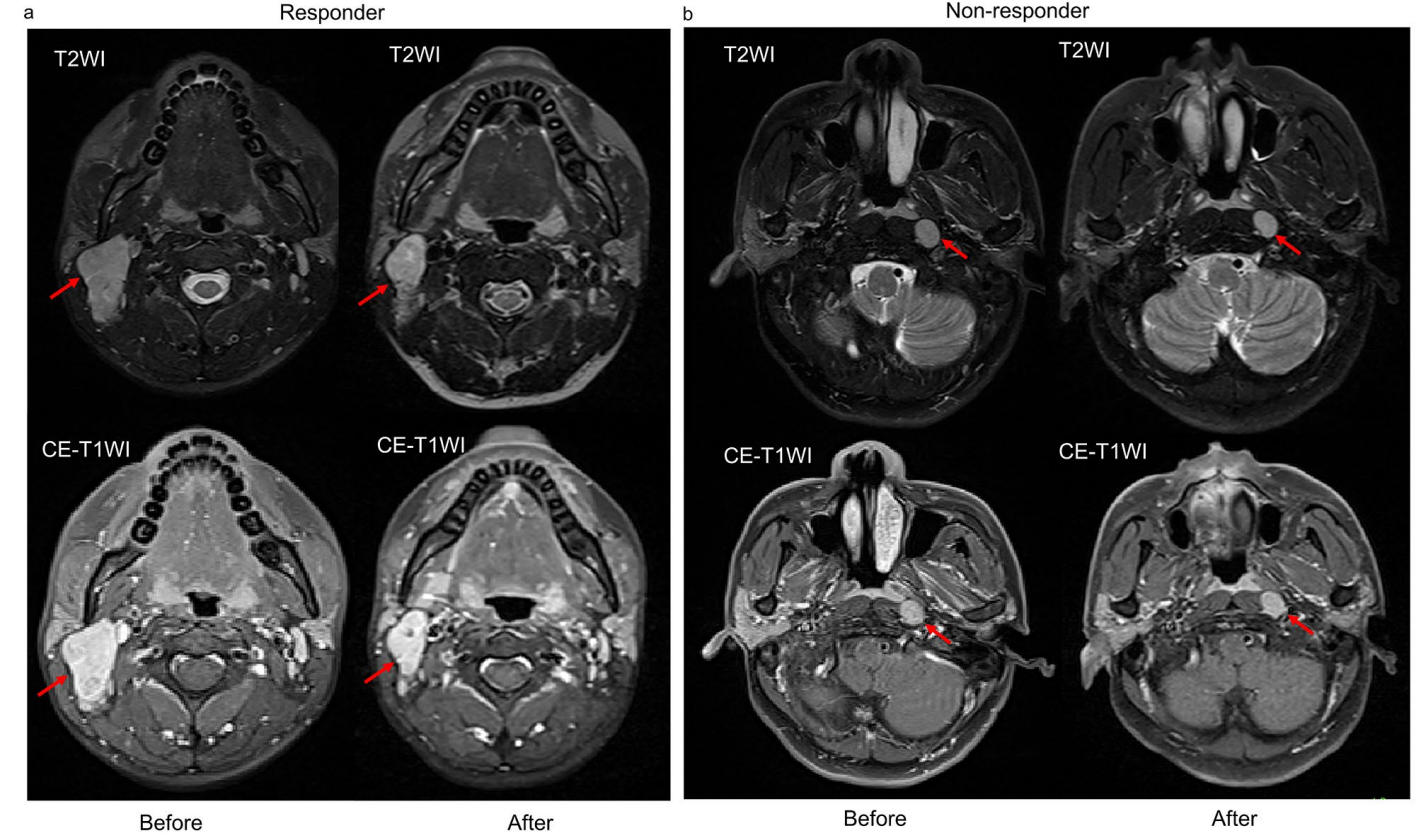
Representative slice of T2WI and CE-T1WI of metastatic cervical lymph nodes (red arrows) before and after radiochemotherapy. (**a**) A sample in the responder group, Rad-socre of Clinical-radiomic model is 5.739, and the probability to be a responder is 0.996; (**b**) A sample in the non-responder group, Rad-socre of Clinical-radiomic model is -4.037, and the probability to be a responder is 0.017. The threshold of probability is 0.481. CE-T1WI, contrast-enhanced T1-weighted imaging; T2WI, T2-weighted imaging

Four predicting models including a clinical-radiomic model and radiomic model of Intra, Peri, and Intra + Peri, were ultimately constructed. The AUCs of the radiomic model of Intra, Peri, Intra + Peri, and Clinical-radiomic were 0.910, 0.887, 0.934, and 0.941, respectively, in the training set and 0.737, 0.794, 0.774, and 0.783, respectively, in the validation set (Table 3). The discriminating performance of the clinical-radiomic model was superior to that of the clinical factor (GTV-ln) in the training set (0.941 vs. 0.709, *P* < 0.001) but not in the validation set (0.783 vs. 0.678, *P* = 0.356). The ROC curves of the radiomic models are shown in Fig. 4. The AUC, Sen, Spe, and ACC of each radiomic model in both cohorts are listed in Table 3. Representative CE-T1WI and T2WI images of NPC patients as responders and non-responders were shown in Fig. 5.

**Table 3:** Diagnostic performance of radiomic models in the training and validation sets

Models	Cohorts	AUC	Sen	Spe	ACC
Intra	Training	0.910 (0.837, 0.958)	0.839 (0.716, 0.923)	0.891 (0.764, 0.963)	0.863 (0.780, 0.922)
	Validation	0.737 (0.580, 0.859)	0.625 (0.405, 0.812)	0.895 (0.668, 0.986)	0.744 (0.588, 0.864)
Peri	Training	0.887 (0.809, 0.941)	0.714 (0.577, 0.827)	0.934 (0.821, 0.986)	0.814 (0.724, 0.883)
	Validation	0.794 (0.643, 0.902)	0.375 (0.187, 0.594)	0.895 (0.668, 0.986)	0.605 (0.444, 0.750)
Intra + Peri	Training	0.934 (0.867, 0.974)	0.946 (0.851, 0.988)	0.782 (0.636, 0.890)	0.873 (0.791, 0.930)
	Validation	0.774 (0.621, 0.887)	0.750 (0.532, 0.902)	0.737 (0.488, 0.908)	0.744 (0.588, 0.864)
Clinical-radiomic	Training	0.941 (0.877, 0.978)	0.929 (0.827, 0.980)	0.848 (0.711, 0.936)	0.892 (0.815, 0.944)
	Validation	0.783 (0.631, 0.894)	0.667 (0.446, 0.843)	0.842 (0.604, 0.966)	0.744 (0.588, 0.864)

The 95% confidence interval was shown in parentheses. AUC the area under the curve, Intra intratumoral, Peri peritumoral, Sen sensitivity, Spe specificity, ACC accuracy

In training and validation sets, the calibration curve of the clinical-radiomic model exhibited excellent agreement between the predictive and observational probability of differentiating between responders and non-responders (Fig. 3b), and the Hosmer-Lemeshow assessment outcomes were non-significant (*P* = 0.618 and 0.780). The Hosmer-Lemeshow test also yielded non-significant results in training and validation sets of the radiomic model of Intra (*P* = 0.405 and 0.165), radiomic model of Peri (*P* = 0.300 and 0.512), and radiomic model of Intra + Peri (*P* = 0.610 and 0.158), which revealed no departure from the ideal fit (Additional file 1: Fig. S4).

### Models comparison and TRIPOD

Comparisons of AUC between the Clinical-radiomic model and the other radiomic models in the training and validation sets are listed in Table 4. According to the DeLong test, no significant difference in the AUCs was found across the radiomic models in the training and validation sets (all *P* > 0.05).

**Table 4:** Comparisons of AUC between the Clinical-radiomic model and the other radiomic models in the training and validation sets

Pairwise comparison	AUC	*Z*	*P* ^†^
**Training set**			
Clinical-radiomic	0.941	-	-
Intra + Peri	0.934	0.762	0.446
Intra	0.910	1.107	0.268
Peri	0.887	1.570	0.116
**Validation set**			
Clinical-radiomic	0.783	-	-
Intra + Peri	0.774	0.586	0.558
Intra	0.737	0.639	0.522
Peri	0.794	0.167	0.867

†*P* values were calculated by the DeLong test. AUC the area under the curve, Intra intratumoral, Peri peritumoral

According to the guidelines of the TRIPOD statement, the type of this study belongs to Type 2b. A full list of TRIPOD was provided in Additional file 1 (Additional file 1: Table S7).

## Discussion

Our results showed that the pretreatment MRI-based radiomics model may be useful in predicting the treatment response of metastatic lymph nodes of NPC. Although the Peri radiomics model does not provide statistically significant incremental value over the Intra model, the generalization ability of the radiomic model of Peri was better than that of Intra and Intra + Peri.

NPC is highly susceptible to regional lymph node metastasis [36]. Additionally, earlier findings indicated that the characteristics of metastatic lymph nodes were indicators of distant metastasis and had an effect on prognosis for overall, local recurrence, regional relapse, and disease-free survival [37–39]. For patients with NPC, due to different levels of sensitivity to the treatment, patients at the same stage may have different treatment responses. In our study, among the 145 included patients, 80 were identified as responders, and 65 were non-responders. The responders and non-responders were 55.2% and 44.8%. The results of our study are comparable to other series [18, 20, 40, 41]. Successful prevention of therapeutic side effects and disease control, however, requires careful consideration of radiotherapy dose and chemotherapy regimen. Using our predictive model, physicians can preliminarily predict the patients’ treatment response and take preventative measures. Based on our result, increasing the GTV-ln could be an effective alternative treatment for these potentially poor responders. Moreover, radiotherapy and chemotherapy are the most common treatments for locoregionally progressed NPC [8]. Early identification of non-responders using our prediction model could aid in making timely and appropriate adjustments to therapy regimens, resulting in considerable clinical benefits for NPC patients, and avoiding unnecessary radiochemotherapy-related side effects.

MRI has been widely utilized to assess treatment response in NPC [42]. No ideal biomarkers, however, were found to predict the treatment response in NPC nowadays. Lu et al.[43] showed that intravoxel incoherent motion (IVIM) MRI analysis had the potential to predict chemoradiotherapy response of metastatic lymph nodes, but only 27 partial response patients were included in this study, making it more difficult to accurately assess the feasibility of IVIM. In addition, there are many MRI-radiomic studying the treatment response in NPC [18, 44–46]. These studies, however, have mainly focused on the primary tumor of NPC. Different from these studies that focus on primary tumors, the present study considered the metastatic lymph node as the ROI. Previous studies have shown that tumor recurrence and distant metastasis are strongly related to the peritumoral microenvironment [47–50]. Microenvironment affects the metastatic potential and the therapeutic effect [51]. To the best of our knowledge, treatment response prediction of NPC using peri-tumoral radiomics features from metastatic lymph nodes has not been investigated. Although the differences in the value of AUCs across the radiomic models were not significant in both sets (all *P* > 0.05; Table 4), we found that the calibration curve of the radiomic model of Peri exhibited excellent agreement between the predictive and observational in both the training and validation sets (Additional file 1: Fig. S4), which indicated the generalization ability of the radiomic model of Peri was better than that of Intra and Intra + Peri through visual inspection. But further research is necessary before these findings can be applied clinically.

Shi et al. have suggested that the involvement of retropharyngeal and cervical may be a potential prognostic factor for NPC [52]. However, in our study, there was no significant difference in lymph node involvement between responders and non-responders in the training and validation sets. It may be the cause of the distribution of lymph node involvement in responders and non-responders groups were not well balanced. Furthermore, numerous studies have confirmed the potential utility of MRI-based lymph nodes characteristics in NPC prognosis assessment, such as nodal grouping, extranodal extension, nodal laterality, and lymph node necrosis [39, 53–55]. The results of these studies provide a good idea for our further research.

Intensity-based features, also known as firstorder features, describe properties of the intensity distribution within a ROI while ignoring the spatial location of each voxel. Besides, Laplacian of Gaussian filter, which emphasize edges region of rapid change, was used in the study. In the radiomic model of Intra + Peri, the firstorder_3D.90Percentile from the Intra region and firstorder_3D.Minimum from the Peri region was negatively connected with responders, exhibiting that the non-responders had a more clear boundary than the responders. The three selected texture features for the radiomic model of Intra + Peri were wavelet features, which can be used to comprehensively quantify tumor heterogeneity across different spatial scales at different directional orientations. The significant gray level co-occurrence matrix feature included MaximumProbability from the Intra region. The non-responders had a higher Maximum Probability, indicating that non-responders have coarser texture and greater tumor heterogeneity in the Intra region. Besides, the remaining two texture features (short-run elevated gray level emphasis, long-run reduced gray level emphasis) of gray level run length matrix from the Peri region were also selected to develop the radiomic model of Intra + Peri. Our findings indicated that, compared to non-responders, the responders showed a larger joint distribution of shorter run lengths with greater gray-level values and long run lengths with lower gray-level values inside the Peri region, indicating that responders have coarser texture and greater tumor heterogeneity in the Peri region.

The current study had several limitations. Firstly, our study was a retrospective analysis, and several essential clinical variables, such as plasma Epstein-Barr virus DNA, were not included in our study as a result of missing or unavailable data. More clinical data need to be incorporated to further improve the model’s discrimination ability. Besides, the time all patients who underwent MRI examination after the completion of treatment was inconsistent, which might have introduced biases and impacted our results. Secondly, the heterogeneity in acquisition parameters of two different magnetic field strength scanners may affect the image texture. Thus, additional studies may be required to quantitatively investigate these effects. In addition, other MRI method, especially DCE-MRI-based radiomic, has been employed in predicting treatment response for breast malignancy and their function in NPC needs to be further explored [56]. Thirdly, the single-center nature and small sample size of our study limit the generalizability of our models. Hence, a multi-center study with large sample size is needed.

In summary, the pretreatment MRI-based radiomics model may be useful in predicting the treatment response of metastatic lymph nodes of NPC. Besides, the generalization ability of the radiomic model of Peri was better than that of Intra and Intra + Peri.

## Electronic supplementary material

Below is the link to the electronic supplementary material.

Supplementary Material 1

## Data Availability

The datasets used and/or analyzed during the current study are available from the corresponding author on reasonable request.

## Abbreviations

AUC: Area under the curve
AJCC: American joint committee on cancer
CI: Confdence internal
CCRT: Concurrent chemoradiotherapy
CT: Computed tomography
CE-T1WI: Contrast-enhanced T1-weighted imaging
GTV-ln: Lymph node gross tumor volume
Intratumoral: Intra
IC: Induction chemotherapy
IMRT: Intensity-modulated radiotherapy
ICC: Intraclass correlation coefficient
IBSI: Image biomarker standardisation initiative
LASSO: Least absolute shrinkage and selection operator
mRMR: Maximum relevance-minimum redundancy
NPC: Nasopharyngeal cancer
NCCN: National comprehensive cancer network
Peritumoral: Peri
RECIST: Response evaluation criteria in solid tumors
ROI: Region of interest
Rad-score: Radiomics score
ROC: Receiver operating characteristic curve
T2WI: T2-weighted imaging
TNM: Tumor-node-metastasis
TRIPOD: Transparent reporting of a multivariable prediction model for individual prognosis or diagnosis.
