# Supplementary material for: Intra- and peritumoral MRI radiomics assisted in predicting radiochemotherapy response in metastatic cervical lymph nodes of nasopharyngeal cancer
Source: BMC Med Imaging. 2023 May 30;23:66. doi: 10.1186/s12880-023-01026-1 (PMC10230802; doi:10.1186/s12880-023-01026-1)
Supplement: Supplementary file 1 — Supplementary Material 1 [file 12880_2023_1026_MOESM1_ESM.docx]

***Supplementary Materials***

***S1:* Treatment regimen**

The doses for the target area was GTV-ln 9–72 Gy. IC drugs were as follows. It consisted of 2-4 cycles of TP regimen (Docetaxel 75 mg/m2 IV in d1, cisplatin 75-100 mg/m2/ d IV for 3 days) or GP regimen (gemcitabine 1000 mg/m2 IV in d1 and d8, cisplatin 75-100mg/m2/ d IV for 3 days) at a 2 week interval prior to the initiation of RT treatment. The CCRT drug was cisplatin (75 mg/m2, d1–3) given every 3 weeks.

***S2:* MRI acquisition parameters**

The 1.5T MRI (Avanto, Siemens, Germany) acquisition parameters were as follows: (1) axial T2-weighted spin-echo images, repetition time (TR)/echo time (TE) = 3280-8290 ms/72-95 ms, slice thickness = 3-4 mm, spacing between slices = 3.6-7.44 mm, echo train length (ETL) = 13-14, number of excitation (NEX) = 1-3, flip angle (FA) = 150°, acquisition type = 2D, in-plane resolution = 0.33 × 0.33 mm^2^ to 0.78 × 0.78 mm^2^; (2) axial contrast-enhanced T1-weighted spin-echo images, TR/TE = 610-1090 ms/11-19 ms, slice thickness = 3 mm, spacing between slices = 3.3-4.5 mm, ETL = 2-3, NEX = 1-2, FA = 138-150°, acquisition type = 2D, in-plane resolution = 0.39 × 0.39 mm^2^ to 1.17 × 1.17 mm^2^ ; (3) axial contrast- enhanced T1-weighted gradient-echo images, TR/TE = 306 ms/4.76 ms, slice thickness = 3 mm, spacing between slices = 3.3 mm, ETL = 1, NEX = 1, FA = 80°, acquisition type = 2D, in-plane resolution = 0.46 × 0.46 mm^2^; (4) axial contrast-enhanced T1-weighted gradient-echo images, TR/TE: 5.95 ms/2.76 ms, slice thickness = 3 mm, spacing between slices = 0 mm, ETL = 1, NEX = 1, FA =10°, acquisition type = 3D, in-plane resolution = 0.98 × 0.98 mm^2^.

The 3.0T MRI (Skyra, Siemens, Germany) acquisition parameters were as follows: (1) axial T2-weighted spin-echo images, TR/TE = 3720-4320 ms/80 ms, slice thickness = 3-4 mm, spacing between slices = 3.6-6.2 mm, ETL = 19, NEX = 1, FA = 168-176°, acquisition type = 2D, in-plane resolution = 0.39 × 0.39 mm^2^ to 0.93 × 0.93 mm^2^; (2) axial contrast- enhanced T1-weighted spin-echo images, TR/TE = 760-4340 ms/11-80 ms, slice thickness = 3-4 mm, spacing between slices= 3.6-4.8 mm, ETL = 4-19, NEX = 1, FA = 142-180°, acquisition type = 2D, in-plane resolution = 0.39 × 0.39 mm^2^ to 0.87 × 0.87 mm^2^; (3) axial contrast- enhanced T1-weighted gradient-echo images, TR/TE = 3.97-4.64 ms/1.29-2.19 ms, slice thickness = 3 mm, spacing between slices = 0-3.3 mm, ETL = 1-2, NEX = 1-2, FA = 9-10°, acquisition type = 3D, in-plane resolution = 0.45 × 0.45 mm^2^ to 1.18 × 1.18 mm^2^.

***Table S1:* Overview of 104 original radiomic features.**

| **Category** | **Features** | **Counts** |
| --- | --- | --- |
| Shape | Elongation, Flatness, Least axis length, Major axis length, Maximum 2D diameter (column), Maximum 2D diameter (row), Maximum 2D diameter (slice), Maximum 3D diameter, Mesh volume, Minor axis length, Sphericity, Surface area, Surface area to volume ratio, Voxel volume | 14 |
| First order | 10 Percentile, 90 Percentile, Energy, Entropy, Interquartile range, Kurtosis^a^, Maximum, Mean absolute deviation, Mean, Median, Minimum, Range, Robust mean absolute deviation, Root mean squared, Skewness, Total energy^b^, Uniformity, Variance | 18 |
| Texture |  |  |
| GLCM | Autocorrelation, Cluster prominence, Cluster shade, Cluster tendency, Contrast, Correlation, Difference average, Difference entropy, Difference variance, Inverse difference normalized (Idn), Inverse difference (Id), Inverse difference moment normalized (Idmn), Inverse difference moment (Idm), Informational measure of correlation 1 (Imc1), Informational measure of correlation 2 (Imc2), Inverse variance, Joint average, Joint energy, Joint entropy, Maximum probability, Sum entropy | 21 |
| GLRLM | Gray level non-uniformity (GLN), Gray level non-uniformity normalized (GLNN), Gray level variance (GLV), High gray level run emphasis (HGLRE), Long run emphasis (LRE), Long run high gray level emphasis (LRHGLE), Long run low gray level emphasis (LRLGLE), Low gray level run emphasis (LGLRE), Run entropy (RE), Run length non-uniformity (RLN), Run length non-uniformity normalized (RLNN), Run percentage (RP), Run variance (RV), Short run emphasis (SRE), Short run high gray level emphasis (SRHGLE), Short run low gray level emphasis (SRLGLE) | 16 |
| GLSZM | Gray level non-uniformity (GLN), Gray level non-uniformity normalized (GLNN), Gray level variance (GLV), High gray level zone emphasis (HGLZE), Large area emphasis (LAE), Large area high gray level emphasis (LAHGLE), Large area low gray level emphasis (LALGLE), Low gray level zone emphasis (LGLZE), Size zone non-uniformity (SZN), Size zone non-uniformity normalized (SZNN), Small area emphasis (SAE), Small area high gray level emphasis (SAHGLE), Small area low gray level emphasis (SALGLE), Zone entropy (ZE), Zone percentage (ZP), Zone variance (ZV) | 16 |
| GLDM | Dependence entropy (DE), Dependence non-uniformity (DN), Dependence non-uniformity normalized (DNN), Dependence variance (DV), Gray level non-uniformity (GLN), Gray level variance (GLV), High gray level emphasis (HGLE), Large dependence emphasis (LDE), Large dependence high gray level emphasis (LDHGLE), Large dependence low gray level emphasis (LDLGLE), Low gray level emphasis (LGLE), Small dependence emphasis (SDE), Small dependence high gray level emphasis (SDHGLE), Small dependence low gray level emphasis (SDLGLE) | 14 |
| NGTDM | Busyness, Coarseness, Complexity, Contrast, Strength | 5 |

GLCM, gray-level cooccurrence matrix; GLRLM, gray-level run length matrix; GLSZM, gray level size zone matrix; GLDM, gray level dependence matrix; NGTDM, neighbouring gray tone difference matrix; IBSI, Imaging Biomarker Standardisation Initiative

^a^ The kurtosis is not corrected by -3, yielding a value 3 higher than the IBSI kurtosis

^b^ Total energy is not present in IBSI feature definitions

***Table S2:* Computation parameters of texture features.**

| **Category** | **Matrix aggregation** | **Distance weighting** | **Chebyshev distance** | **Other** |
| --- | --- | --- | --- | --- |
| GLCM | ITBB^a^ | no weighting | 1 | symmetric matrix |
| GLRLM | ITBB^a^ | no weighting | - | - |
| GLSZM | KOBO^b^ | - | 1 | - |
| GLDM | KOBO^b^ | no weighting | 1 | coarseness parameter α =0 |
| NGTDM | KOBO^b^ | no weighting | 1 | - |

GLCM, gray-level cooccurrence matrix; GLRLM, gray-level run length matrix; GLSZM, gray level size zone matrix; GLDM, gray level dependence matrix; NGTDM, neighbouring gray tone difference matrix; IBSI, Imaging Biomarker Standardisation Initiative

^a^ Features are computed from each 3D directional matrix and averaged over the 3D directions (IBSI code ITBB)

^b^ The feature is computed from a 3D matrix (IBSI code KOBO)

***Table S3:* Results and description of the radiomic features selected.**

| **Radiomic model** | **Image** | **Filter** | **Feature class** | **Feature*** |
| --- | --- | --- | --- | --- |
| **Intra** | CE-T1WI | Wavelet (HHH) | Firstorder**†** | Skewness |
|  | CE-T1WI | Wavelet (HHL) | GLDM**‡** | Large Dependence Low Gray Level Emphasis |
|  | T2WI | **♮**Log (sigma = 1.5) | GLCM**§** | Cluster Shade |
|  | T2WI | **‖**Wavelet (LLH) | GLCM | MaximumProbability |
| **Peri** | CE-T1WI | Log (sigma = 0.5) | Firstorder | Minimum |
|  | CE-T1WI | Wavelet (HHH) | GLRLM**¶** | Short Run High Gray Level Emphasis |
|  | T2WI | Wavelet (LHL) | GLDM | Small Dependence High Gray Level Emphasis |
|  | T2WI | Wavelet (LLH) | GLCM | ClusterTendency |
| **Intra + Peri** | CE-T1WI | Log (sigma = 0.5) | Firstorder | 90Percentile |
|  | CE-T1WI | Log (sigma = 0.5) | Firstorder | Minimum |
|  | CE-T1WI | Wavelet (HHH) | GLRLM | Short Run High Gray Level Emphasis |
|  | T2WI | Wavelet (LLH) | GLCM | MaximumProbability |
|  | T2WI | Wavelet (HLL) | GLRLM | Long Run Low Gray Level Emphasis |

CE-T1WI, contrast-enhanced T1-weighted imaging; T2WI, T2-weighted imaging; Intra, intratumoral; Peri, peritumoral; GLCM, gray-level cooccurrence matrix; GLDM, gray level dependence matrix; GLRLM, gray-level run length matrix

**†** Firstorder: First-order statistics describe the distribution of voxel intensities within the image region defined by the mask through commonly used and basic metrics.

**‡** Gray Level Dependence Matrix (GLDM): A Gray Level Dependence Matrix (GLDM) quantifies gray level dependencies in an image. A gray level dependency is defined as a the number of connected voxels within distance δ that are dependent on the center voxel.

**§** Gray Level Co-occurrence Matrix (GLCM): A Gray Level Co-occurrence Matrix of size Ng×Ng describes the second-order joint probability function of an image region constrained by the mask and is defined as P(i,j|δ,θ).

**¶** Gray Level Run Length Matrix (GLRLM): A Gray Level Run Length Matrix (GLRLM) quantifies gray level runs, which are defined as the length in number of pixels, of consecutive pixels that have the same gray level value. In a gray level run length matrix P(i,j|θ), the (i,j)th element describes the number of runs with gray level i and length j occur in the image (ROI) along angle θ.

**♮** The Gaussian kernel is used to smooth the image and is defined as:

$$G\left(x,y,z,\sigma\right)=\frac{1}{\left(\sigma\sqrt{2\pi}\right)^{3}}e^{-\frac{x^{2}+y^{2}+z^{2}}{2\sigma^{2}}}$$

The Gaussian kernel is convolved by the laplacian kernel $\nabla^{2}G\left(x,y,z\right)$, which is sensitive to areas with rapidly changing intensities, enhancing edges. The width of the filter in the Gaussian kernel is determined by σ. In this study, σ was set to 0.5, 1.0, 1.5, and 2.0 mm.

**‖** The Coiflets wavelet (coif1) is applied on images in this study. The original image *X* is decomposed into 8 decompositions by directional low-pass (i.e. a scaling) and high-pass (i.e. a wavelet) filtering. The *L* denotes low-pass and *H* denotes high-pass. The wavelet decompositions of *X* are labeled as *X_LLL_*, *X_LLH_, X_LHL_, X_LHH_, X_HLL_, X_HLH_, X_HHL_,* and *X_HHH_*, according to the respective ordering of low or high-pass filtering in x, y and z direction.

***** The descriptions and feature explanations of all these features can be found on the official "PyRadiomics" website (https://pyradiomics.readthedocs.io).

***Table S4:* Multivariate logistic regression of raidomic features for differentiating responders from non-responders in the training set.**

| **Radiomic model** | **Radiomic features** | **Odds ratio** | **95% CI** | ***P*** |
| --- | --- | --- | --- | --- |
| **Intra** | CE-T1WI_firstorder_wavelet.HHH.Skewness | 0.420 | 0.214-0.823 | 0.011 |
|  | CE-T1WI_GLDM_wavelet.HHL.LDLGLE | 3.393 | 1.659-6.938 | 0.001 |
|  | T2WI_GLCM_LoG.sigma.1.5.mm.3D.Cluster  Shade | 16.768 | 3.614-77.806 | < 0.001 |
|  | T2WI_GLCM_wavelet.LLH.MaximumProbability | 0.112 | 0.023-0.537 | 0.006 |
| **Peri** | CE-T1WI_firstorder_LoG.sigma.0.5.mm.3D.  Minimum | 0.271 | 0.122-0.602 | 0.001 |
|  | CE-T1WI_GLRLM_wavelet.HHH.SRHGLE | 6.573 | 2.534-17.051 | < 0.001 |
|  | T2WI_GLDM_wavelet.LHL.SDHGLE | 3.044 | 1.562-5.932 | 0.001 |
|  | T2WI_GLCM_wavelet.LLH.ClusterTendency | 0.259 | 0.130-0.515 | < 0.001 |
| **Intra + Peri** | Intra_CE-T1WI_firstorder_LoG.sigma.0.5.mm.  3D.90Percentile | 0.357 | 0.178-0.714 | 0.004 |
|  | Intra_T2WI_GLCM_wavelet.LLH.  MaximumProbability | 0.049 | 0.009-0.270 | 0.001 |
|  | Peri_CE-T1WI_firstorder_LoG.sigma.0.5.mm.  3D.Minimum | 0.153 | 0.052-0.451 | 0.001 |
|  | Peri_CE-T1WI_GLRLM_wavelet.HHH.  SRHGLE | 3.959 | 1.482-10.573 | 0.006 |
|  | Peri_T2WI_GLRLM_wavelet.HLL.LRLGLE | 4.355 | 1.774-10.694 | 0.001 |

CE-T1WI, contrast-enhanced T1-weighted imaging; T2WI, T2-weighted imaging; Intra, intratumoral; Peri, peritumoral; GLCM, gray-level cooccurrence matrix; GLDM, gray level dependence matrix; GLRLM, gray-level run length matrix; LDLGLE, Large Dependence Low Gray Level Emphasis; SDHGLE, Small Dependence High Gray Level Emphasis; SRHGLE, Short Run High Gray Level Emphasis; LRLGLE, Long Run Low Gray Level Emphasis

***Table S5:* The mean and standard deviation of features in radiomic models.**

| **Features** | **Mean** | **SD** |
| --- | --- | --- |
| **Radiomic model of Intra** |  |  |
| CE-T1WI_firstorder_wavelet.HHH.Skewness | -0.01896 | 0.045360 |
| CE-T1WI_GLDM_wavelet.HHL.LDLGLE | 101.17690 | 7.197824 |
| T2WI_GLCM_LoG.sigma.1.5.mm.3D.ClusterShade | 0.41444 | 0.148076 |
| T2WI_GLCM_wavelet.LLH.MaximumProbability | 0.30155 | 0.017765 |
| **Radiomic model of Peri** |  |  |
| CE-T1WI_firstorder_LoG.sigma.0.5.mm.3D.Minimum | -1.36493 | 0.308836 |
| CE-T1WI_GLRLM_wavelet.HHH.SRHGLE | 1.78029 | 0.010525 |
| T2WI_GLDM_wavelet.LHL.SDHGLE | 0.03263 | 0.002087 |
| T2WI_GLCM_wavelet.LLH.ClusterTendency | 0.52374 | 0.007614 |
| **Radiomic model of Intra + Peri** |  |  |
| Intra_CE-T1WI_firstorder_LoG.sigma.0.5.mm.3D.90Percentile | 0.32375 | 0.053919 |
| Intra_T2WI_GLCM_wavelet.LLH.MaximumProbability | 0.30155 | 0.017765 |
| Peri_CE-T1WI_firstorder_LoG.sigma.0.5.mm.3D.Minimum | -1.36493 | 0.308836 |
| Peri_CE-T1WI_GLRLM_wavelet.HHH. SRHGLE | 1.78029 | 0.010525 |
| Peri_T2WI_GLRLM_wavelet.HLL.LRLGLE | 2.61940 | 0.228950 |

CE-T1WI, contrast-enhanced T1-weighted imaging; T2WI, T2-weighted imaging; SD, standard deviation; Intra, intratumoral; Peri, peritumoral; GLCM, gray-level cooccurrence matrix; GLDM, gray level dependence matrix; GLRLM, gray-level run length matrix; LDLGLE, Large Dependence Low Gray Level Emphasis; SDHGLE, Small Dependence High Gray Level Emphasis; SRHGLE, Short Run High Gray Level Emphasis; LRLGLE, Long Run Low Gray Level Emphasis

***Table S6:* Multicollinearity of the features in the radiomic models.**

| **Feature 1** | **Feature 2** | ***r*** | ***P*** |
| --- | --- | --- | --- |
| **Radiomic model of Intra** | | | |
| CE-T1WI_firstorder_wavelet.HHH.Skewness | CE-T1WI_GLDM_wavelet.HHL.  LDLGLE | - 0.048 | 0.630 |
| CE-T1WI_firstorder_wavelet.HHH.Skewness | T2WI_GLCM_LoG.sigma.1.5.mm.3D.ClusterShade | - 0.101 | 0.311 |
| CE-T1WI_firstorder_wavelet.HHH.Skewness | T2WI_GLCM_wavelet.LLH.MaximumProbability | - 0.059 | 0.554 |
| CE-T1WI_GLDM_wavelet.HHL.LDLGLE | T2WI_GLCM_LoG.sigma.1.5.mm.3D.ClusterShade | 0.348 | < 0.001 |
| CE-T1WI_GLDM_wavelet.HHL.LDLGLE | T2WI_GLCM_wavelet.LLH.MaximumProbability | - 0.296 | 0.003 |
| T2WI_GLCM_LoG.sigma.1.5.mm.3D.ClusterShade | T2WI_GLCM_wavelet.LLH.MaximumProbability | - 0.107 | 0.286 |
| **Radiomic model of Peri** | | | |
| CE-T1WI_firstorder_LoG.sigma.0.5.mm.3D.Minimum | CE-T1WI_GLRLM_wavelet.HHH.  SRHGLE | - 0.025 | 0.800 |
| CE-T1WI_firstorder_LoG.sigma.0.5.mm.3D.Minimum | T2WI_GLDM_wavelet.LHL.SDHGLE | - 0.045 | 0.650 |
| CE-T1WI_firstorder_LoG.sigma.0.5.mm.3D.Minimum | T2WI_GLCM_wavelet.LLH.  ClusterTendency | 0.035 | 0.726 |
| CE-T1WI_GLRLM_wavelet.HHH.SRHGLE | T2WI_GLDM_wavelet.LHL.SDHGLE | 0.093 | 0.351 |
| CE-T1WI_GLRLM_wavelet.HHH.SRHGLE | T2WI_GLCM_wavelet.LLH.Cluster  Tendency | 0.048 | 0.629 |
| T2WI_GLDM_wavelet.LHL.SDHGLE | T2WI_GLCM_wavelet.LLH.Cluster  Tendency | 0.120 | 0.231 |
| **Radiomic model of Intra + Peri** | | | |
| Intra_CE-T1WI_firstorder_LoG.sigma.0.5.mm.3D.90Percentile | Intra_T2WI_GLCM_wavelet.LLH.  MaximumProbability | 0.195 | 0.049 |
| Intra_CE-T1WI_firstorder_LoG.sigma.0.5.mm.3D.90Percentile | Peri_CE-T1WI_firstorder_LoG.sigma.  0.5.mm.3D.Minimum | - 0.084 | 0.399 |
| Intra_CE-T1WI_firstorder_LoG.sigma.0.5.mm.3D.90Percentile | Peri_CE-T1WI_GLRLM_wavelet.HHH. SRHGLE | - 0.188 | 0.059 |
| Intra_CE-T1WI_firstorder_LoG.sigma.0.5.mm.3D.90Percentile | Peri_T2WI_GLRLM_wavelet.HLL.  LRLGLE | - 0.114 | 0.256 |
| Intra_T2WI_GLCM_wavelet.LLH.MaximumProbability | Peri_CE-T1WI_firstorder_LoG.sigma.  0.5.mm.3D.Minimum | - 0.117 | 0.243 |
| Intra_T2WI_GLCM_wavelet.LLH.MaximumProbability | Peri_CE-T1WI_GLRLM_wavelet.HHH. SRHGLE | - 0.219 | 0.027 |
| Intra_T2WI_GLCM_wavelet.LLH.MaximumProbability | Peri_T2WI_GLRLM_wavelet.HLL.  LRLGLE | 0.000 | 0.999 |
| Peri_CE-T1WI_firstorder_LoG.sigma.0.5.mm.3D.Minimum | Peri_CE-T1WI_GLRLM_wavelet.HHH. SRHGLE | - 0.025 | 0.800 |
| Peri_CE-T1WI_firstorder_LoG.sigma.0.5.mm.3D.Minimum | Peri_T2WI_GLRLM_wavelet.HLL.  LRLGLE | - 0.066 | 0.508 |
| Peri_CE-T1WI_GLRLM_wavelet.HHH. SRHGLE | Peri_T2WI_GLRLM_wavelet.HLL.  LRLGLE | 0.187 | 0.060 |

CE-T1WI, contrast-enhanced T1-weighted imaging; T2WI, T2-weighted imaging; Intra, intratumoral; Peri, peritumoral; GLCM, gray-level cooccurrence matrix; GLDM, gray level dependence matrix; GLRLM, gray-level run length matrix; LDLGLE, Large Dependence Low Gray Level Emphasis; SDHGLE, Small Dependence High Gray Level Emphasis; SRHGLE, Short Run High Gray Level Emphasis; LRLGLE, Long Run Low Gray Level Emphasis

***Table S7:* TRIPOD Checklist of this study.**

| **Section/Topic** **Item** **Checklist Item** **Page** | | | | |
| --- | --- | --- | --- | --- |
| **Title and abstract** | | | | |
| Title | 1 | D;V | Identify the study as developing and/or validating a multivariable prediction model, the target population, and the outcome to be predicted. | 1 |
| Abstract | 2 | D;V | Provide a summary of objectives, study design, setting, participants, sample size, predictors, outcome, statistical analysis, results, and conclusions. | Abstract |
| **Introduction** | | | | |
| Background and objectives | 3a | D;V | Explain the medical context (including whether diagnostic or prognostic) and rationale for developing or validating the multivariable prediction model, including references to existing models. | 3-4 |
|  | 3b | D;V | Specify the objectives, including whether the study describes the development or validation of the model or both. | 3-4 |
| **Methods** | | | | |
| Source of data | 4a | D;V | Describe the study design or source of data (e.g., randomized trial, cohort, or registry data), separately for the development and validation data sets, if applicable. | 5 |
|  | 4b | D;V | Specify the key study dates, including start of accrual; end of accrual; and, if applicable, end of follow-up. | 5 |
| Participants | 5a | D;V | Specify key elements of the study setting (e.g., primary care, secondary care, general population) including number and location of centres. | 5 |
|  | 5b | D;V | Describe eligibility criteria for participants. | 5 |
|  | 5c | D;V | Give details of treatments received, if relevant. | Additional file 1 |
| Outcome | 6a | D;V | Clearly define the outcome that is predicted by the prediction model, including how and when assessed. | 6-7 |
|  | 6b | D;V | Report any actions to blind assessment of the outcome to be predicted. | NA |
| Predictors | 7a | D;V | Clearly define all predictors used in developing or validating the multivariable prediction model, including how and when they were measured. | 6-7 |
|  | 7b | D;V | Report any actions to blind assessment of predictors for the outcome and other predictors. | 5-7 |
| Sample size | 8 | D;V | Explain how the study size was arrived at. | 5 |
| Missing data | 9 | D;V | Describe how missing data were handled (e.g., complete-case analysis, single imputation, multiple imputation) with details of any imputation method. | 5; Figure 1 |
| Statistical analysis methods | 10a | D | Describe how predictors were handled in the analyses. | 7-9 |
|  | 10b | D | Specify type of model, all model-building procedures (including any predictor selection), and method for internal validation. | 7-9 |
|  | 10c | V | For validation, describe how the predictions were calculated. | 7-9 |
|  | 10d | D;V | Specify all measures used to assess model performance and, if relevant, to compare multiple models. | 7-9 |
|  | 10e | V | Describe any model updating (e.g., recalibration) arising from the validation, if done. | NA |
| Risk groups | 11 | D;V | Provide details on how risk groups were created, if done. | NA |
| Development vs. validation | 12 | V | For validation, identify any differences from the development data in setting, eligibility criteria, outcome, and predictors. | NA |
| **Results** | | | | |
| Participants | 13a | D;V | Describe the flow of participants through the study, including the number of participants with and without the outcome and, if applicable, a summary of the follow-up time. A diagram may be helpful. | 10 |
|  | 13b | D;V | Describe the characteristics of the participants (basic demographics, clinical features, available predictors), including the number of participants with missing data for predictors and outcome. | 10 |
|  | 13c | V | For validation, show a comparison with the development data of the distribution of important variables (demographics, predictors and outcome). | 10 |
| Model development | 14a | D | Specify the number of participants and outcome events in each analysis. | 10 |
|  | 14b | D | If done, report the unadjusted association between each candidate predictor and outcome. | NA |
| Model specification | 15a | D | Present the full prediction model to allow predictions for individuals (i.e., all regression coefficients, and model intercept or baseline survival at a given time point). | 10-12 |
|  | 15b | D | Explain how to the use the prediction model. | 10-12 |
| Model performance | 16 | D;V | Report performance measures (with CIs) for the prediction model. | 10-12 |
| Model-updating | 17 | V | If done, report the results from any model updating (i.e., model specification, model performance). | NA |
| **Discussion** | | | | |
| Limitations | 18 | D;V | Discuss any limitations of the study (such as nonrepresentative sample, few events per predictor, missing data). | 15-16 |
| Interpretation | 19a | V | For validation, discuss the results with reference to performance in the development data, and any other validation data. | 13-15 |
|  | 19b | D;V | Give an overall interpretation of the results, considering objectives, limitations, results from similar studies, and other relevant evidence. | 13-15 |
| Implications | 20 | D;V | Discuss the potential clinical use of the model and implications for future research. | 13-15 |
| **Other information** | | | | |
| Supplementary information | 21 | D;V | Provide information about the availability of supplementary resources, such as study protocol, Web calculator, and data sets. | Additional file 1 |
| Funding | 22 | D;V | Give the source of funding and the role of the funders for the present study. | 23 |

*Items relevant only to the development of a prediction model are denoted by D, items relating solely to a validation of a prediction model are denoted by V, and items relating to both are denoted D;V.

*NA*,not applicable.


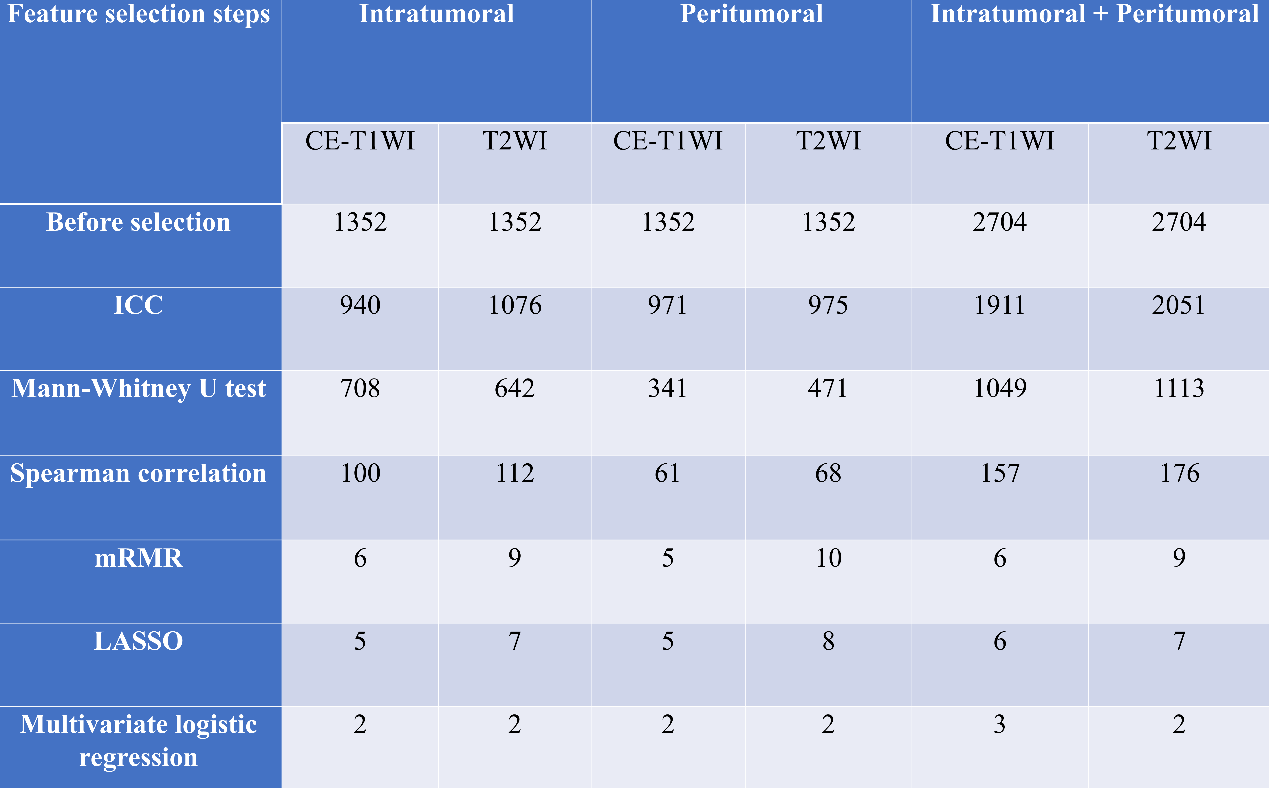
***Fig. S1*** Radiomic feature selection workflow. CE-T1WI, contrast-enhanced T1-weighted imaging; T2WI, T2-weighted imaging; ICC, intraclass correlation coefficients; mRMR, minimum redundancy-maximum relevance; LASSO, least absolute shrinkage and selection operator

***Fig. S2:*** The feature selection process for (**a, b**) Intra features, (**c, d**) Peri features, (**e, f**) and Intra + Peri features based on the LASSO binary logistic regression model. (**a, c, e**) 10-fold cross-validation via one standard error of the minimum criteria (1-SE criteria) in the lasso model is used to select feature by tuning parameter lambda (λ). Binomial deviances from the LASSO regression cross-validation procedure are plotted as a function of log (λ). (**b, d, f**) The optimal λ obtains 12, 13, and 13 nonzero coefficients in Intra, Peri, and Intra + Peri datasets. Intra, intratumoral; Peri, peritumoral; LASSO, least absolute shrinkage and selection operator


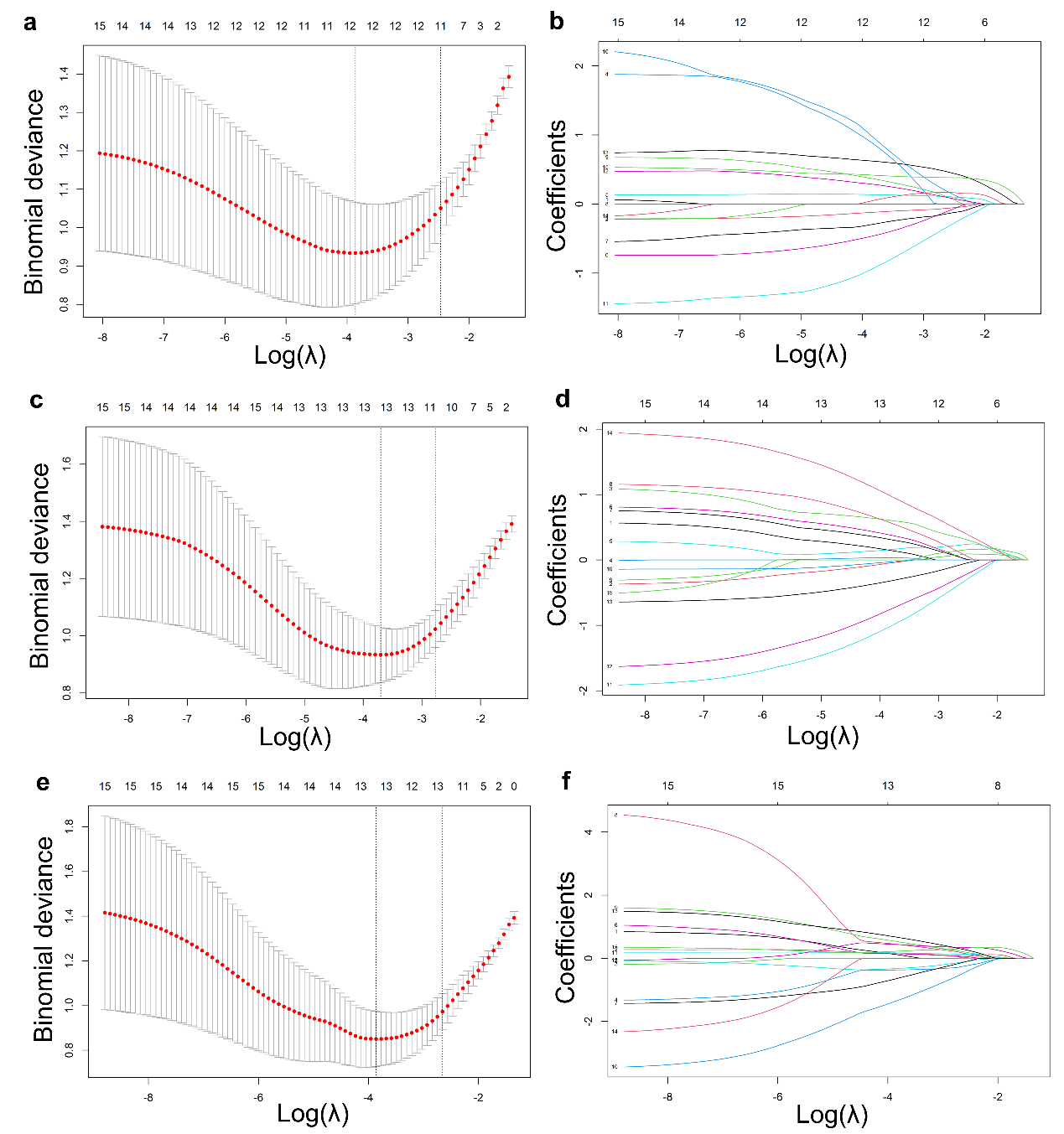


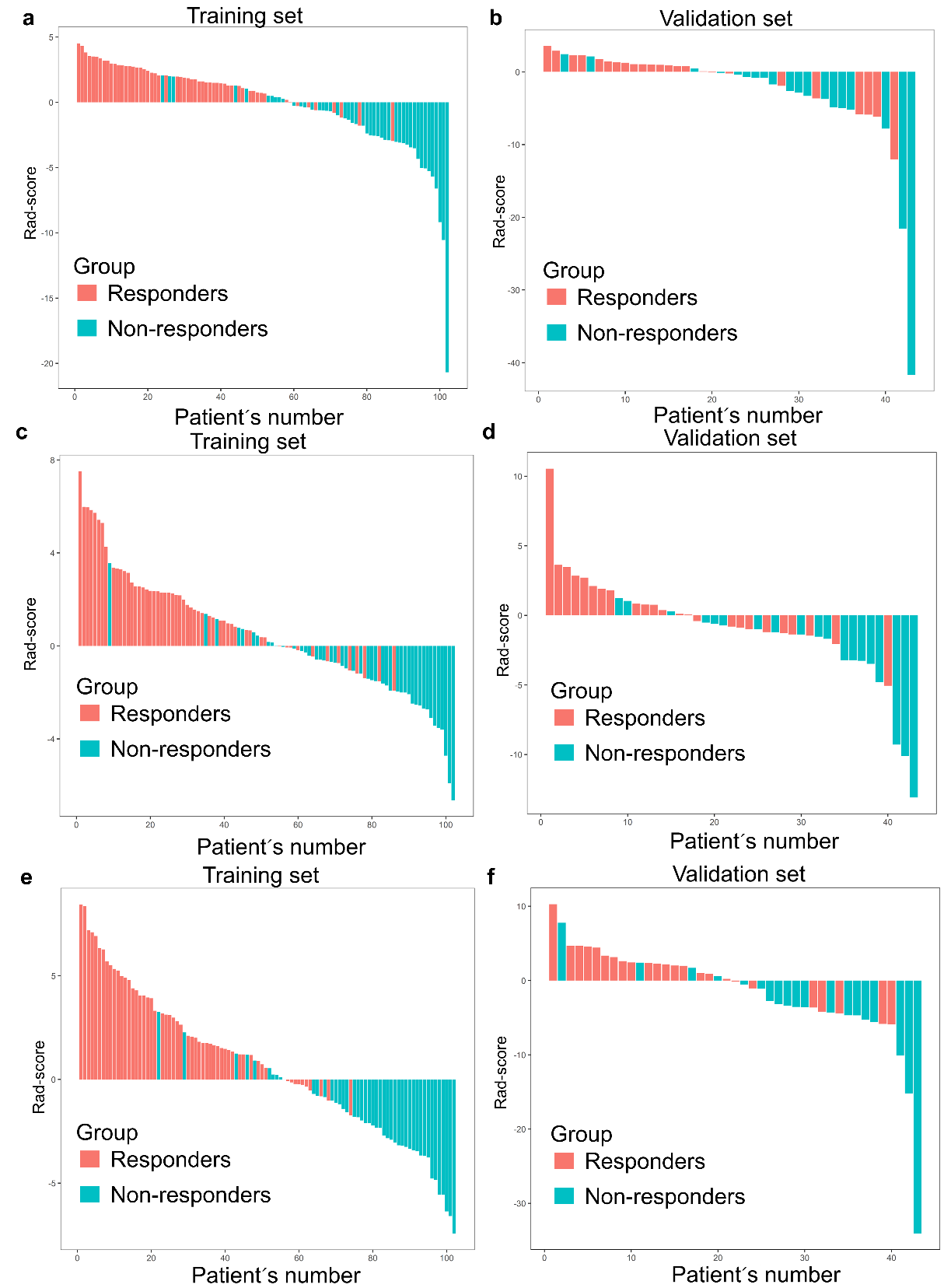
***Fig. S3*** Distributions of Rad-score of responders and non-responders in the training and validation sets. (**a**) Intra_Rad-score in the training set. (**b**) Intra_Rad-score in the validation set. (**c**) Peri_Rad-score in the training set. (**d**) Peri_Rad-score in the validation set. (**e**) Intra + Peri_Rad-score in the training set. (**f**) Intra + Peri_Rad-score in the validation set. Rad-score, radiomics score; Intra, intratumoral; Peri, peritumoral


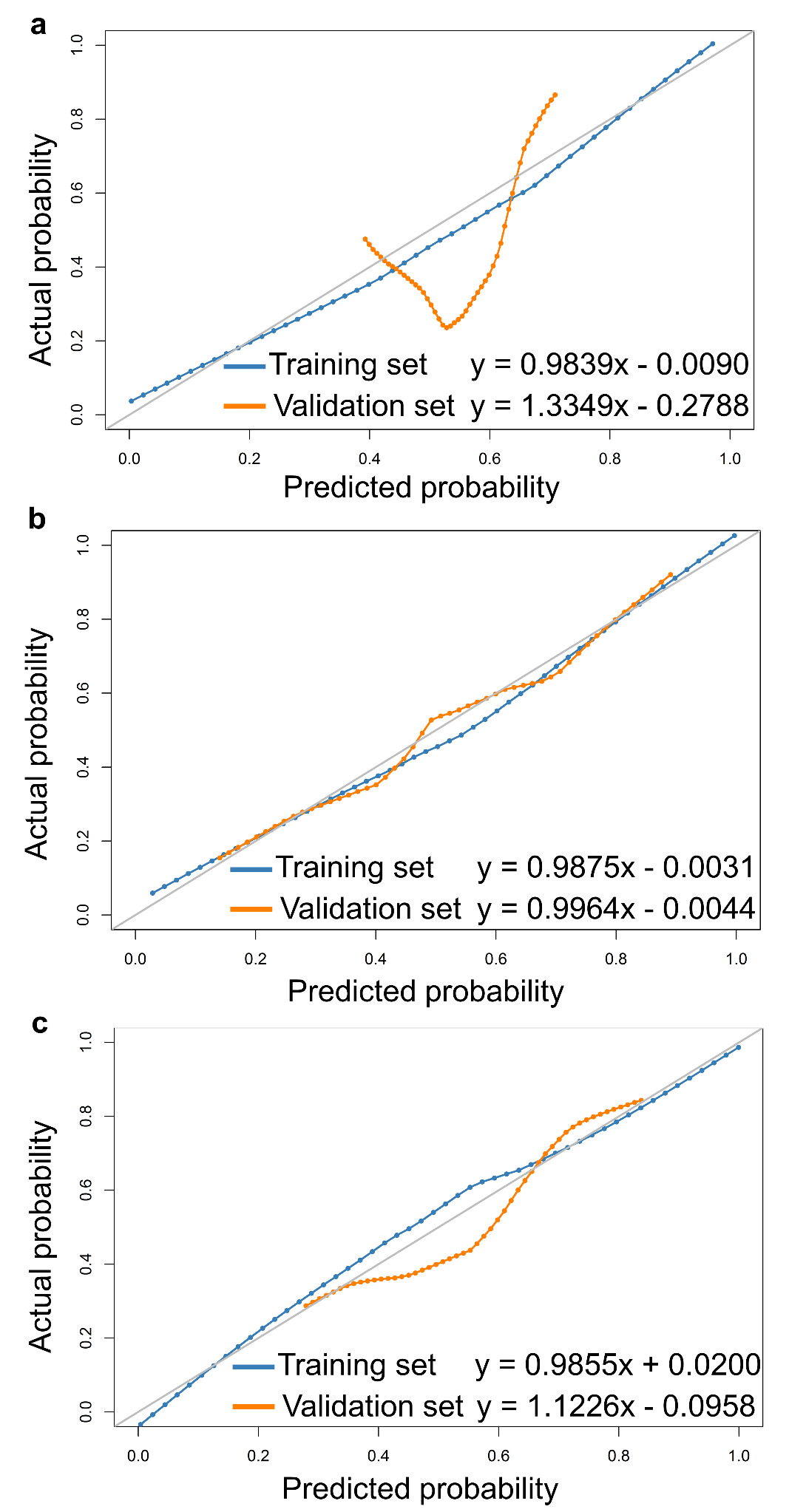
***Fig. S4*** Calibration curves of the radiomic models. (**a**) Radiomic model of Intra. (**b**) Radiomic model of Peri. (**c**) Radiomic model of Intra + Peri. Intra, intratumoral; Peri, peritumoral
